# FDK Half-Scan with a Heuristic Weighting Scheme on a Flat Panel Detector-Based Cone Beam CT (FDKHSCW)

**DOI:** 10.1155/IJBI/2006/83983

**Published:** 2006-09-05

**Authors:** Dong Yang, Ruola Ning

**Affiliations:** Department of Imaging Sciences and Electrical & Computer Engineering, University of Rochester Medical Center, Rochester, NY 14642, USA

## Abstract

A cone beam circular half-scan scheme is becoming an attractive imaging method in cone beam CT since it improves the temporal resolution. Traditionally, the redundant data in the circular half-scan range is weighted by a central scanning plane-dependent weighting function; FDK algorithm is then applied on the weighted projection data for reconstruction. However, this scheme still suffers the attenuation coefficient drop inherited with FDK when the cone angle becomes large. A new heuristic cone beam geometry-dependent weighting scheme is proposed based on the idea that there exists less redundancy for the projection data away from the central scanning plane. The performance of FDKHSCW scheme is evaluated by comparing it to the FDK full-scan (FDKFS) scheme and the traditional FDK half-scan scheme with Parker's fan beam weighting function (FDKHSFW). Computer simulation is employed and conducted on a 3D Shepp-Logan phantom. The result illustrates a correction of FDKHSCW to the attenuation coefficient drop in the off-scanning plane associated with FDKFS and FDKHSFW while maintaining the same spatial resolution.


Hindawi Publishing Corporation
International Journal of Biomedical Imaging
Volume 2006, Article ID 83983, Pages 1–8
DOI 10.1155/IJBI/2006/83983

### FDK Half-Scan with a Heuristic Weighting Scheme on a Flat Panel Detector-Based Cone Beam CT (FDKHSCW)

**Dong Yang and Ruola Ning**

*Department of Imaging Sciences and Electrical & Computer Engineering, University of Rochester Medical Center, Rochester, NY 14642, USA*

Received 1 December 2005; Revised 13 June 2006; Accepted 17 June 2006

A cone beam circular half-scan scheme is becoming an attractive imaging method in cone beam CT since it improves the temporal resolution. Traditionally, the redundant data in the circular half-scan range is weighted by a central scanning plane-dependent weighting function; FDK algorithm is then applied on the weighted projection data for reconstruction. However, this scheme still suffers the attenuation coefficient drop inherited with FDK when the cone angle becomes large. A new heuristic cone beam geometry-dependent weighting scheme is proposed based on the idea that there exists less redundancy for the projection data away from the central scanning plane. The performance of FDKHSCW scheme is evaluated by comparing it to the FDK full-scan (FDKFS) scheme and the traditional FDK half-scan scheme with Parker's fan beam weighting function (FDKHSFW). Computer simulation is employed and conducted on a 3D Shepp-Logan phantom. The result illustrates a correction of FDKHSCW to the attenuation coefficient drop in the off-scanning plane associated with FDKFS and FDKHSFW while maintaining the same spatial resolution.

Copyright © 2006 D. Yang and R. Ning. This is an open access article distributed under the Creative Commons Attribution License, which permits unrestricted use, distribution, and reproduction in any medium, provided the original work is properly cited.

### 1. INTRODUCTION

The use of the half-scan method in cone beam CT has been a hot topic in the recent years owing to the resultant improvement in temporal resolution [1, 2]. There are currently several different types of cone beam half-scan schemes, such as FDK-based [3–5], cone beam filtered-backprojection-based (CBFBP) [6], and Grangeat-based [7]. Each scheme uses planar scanning trajectories (circular or noncircular) to conduct the half-scan scheme. Theoretically, a circular half scan can acquire approximately the same information in the radon domain as a circular full scan in terms of the first derivative radial data, as long as the reconstructed object is within a certain size based on the derivation of the Grangeat formula [8]. Even in the circular half-scanning range, redundancy still exists. The Grangeat-type half scan (GHS) maps the spatial projection data into the first derivative radial data and weights them in the radon domain. After adding missing data through linear interpolation/extrapolation in the shadow zone of the radon domain where a circular scan cannot access, a 3D radon inverse formula is used to get the reconstructed image.

Current FDK-type half-scan (FDKHSFW) schemes for cone beam CT use Parker's [9] or other weighting coefficients based on fan beam geometry, where same weighting coefficients are applied to all detector rows. The CBFBP algorithm manipulates the redundant projection data in the radon domain; does the half-scan reconstruction in the structure of filtered backprojection (FBP) and achieves almost the same performance as FDKHSFW. The Grangeat-type half-scan scheme outperforms the FDK-type half-scan scheme in the correction of the off-scanning plane attenuation coefficient drop when the shadow zone is filled with the linear interpolated data. However, the spatial resolution of the reconstructed images from GHS is inferior to that of FDKHSFW because data interpolation is less involved in FDK than in GHS [10]. Furthermore, GHS cannot handle the truncated data in the longitudinal direction. The CBFBP-related half-scan and FDKHSFW showed obvious attenuation coefficient drop artifacts in the position of the reconstructed image farthest away from $Z = 0$, where $Z$ is the rotation axis. This artifact is undesirable in practice.

In order to correct this drop problem to a certain degree as well as to maintain spatial resolution, we propose an FDK half-scan scheme with a new weighting function that fits the cone beam geometry (FDKHSCW), where the weighting function is cone beam geometry dependent. In Section 2, the FDK half-scan algorithm with the new cone beam weighting

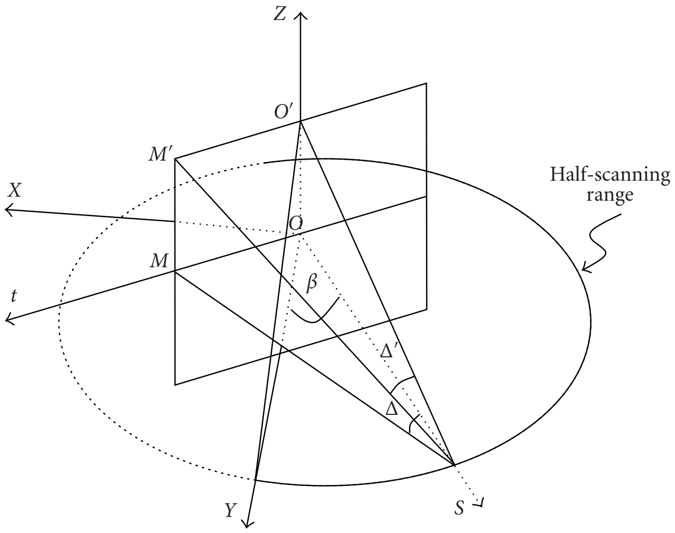

Figure 1: Equal space cone beam geometry with circular scan.

function is described. In Section 3, the computer simulation is conducted and the FDKHSCW is evaluated in comparison to FDKFS and FDKHSFW. Discussions and conclusions are included in Section 4.

### 2. CIRCULAR CONE BEAM HALF-SCAN SCHEME (FDKHSCW)

### 2.1. Cone beam half-scan weighting function

The FDK [11] algorithm expands upon the fan beam algorithm by summing the contribution to the object of all the tilted fan beams. The reconstruction is based on filtering and back projecting a single fan beam within the cone. Based on the cone beam geometry in Figure 1, the formula of the FDK is

$$f(x,y,z) = \frac{1}{2}\int_0^{2\pi} \frac{so^2}{(so-s)^2} \cdot \left\{\left[R_\beta(np,m\xi)\frac{so}{\sqrt{so^2+m^2\xi^2+n^2p^2}}\right] * h(np)\right\} d\beta,$$

$$s = -x\sin\beta + y\cos\beta. \tag{1}$$

The $*$ sign denotes the convolution; $so$ the distance from the X-ray source to the origin; $n$, $m$ the integer value where $n = 0$ and $m = 0$ correspond to the central ray passing through the origin; $\beta$ the projection angle defined in the scanning plane; $p$ the virtual detector sampling interval along the $t$ axis; $\xi$ the virtual detector sampling interval along the $Z$ axis; $R_\beta(np, m\xi)$ the actual discrete 2D projection data; and $h(np)$ the discrete one-dimensional ramp filter impulse response along the $t$ axis.

The preweight term, $so/\sqrt{so^2 + m^2\xi^2 + n^2p^2}$, can be factorized into two cosine terms as

$$\left(\frac{\sqrt{so^2+n^2p^2}}{\sqrt{so^2+m^2\xi^2+n^2p^2}}\right)\left(\frac{so}{\sqrt{so^2+n^2p^2}}\right). \tag{2}$$

This means that FDK projects the off-scanning plane projection data into the scanning plane and then follows the 2D fan beam reconstruction algorithm. In (1), the factor of 1/2 in front of the integral is used to cancel the projection redundancy when a full circular scanning is conducted. This implies that the off-scanning plane projection data has the same redundancy as the projection data in the scanning plane.

Cone beam half-scan scheme is also the extension of the fan beam half scan combined with the FDK, in which the weighting coefficients calculated from the scanning plane geometry are applied to all projection rows as follows:

$$f(x,y,z) = \int_0^{\pi+2\Delta} \frac{so^2}{(so-s)^2} \cdot \left\{\left[\omega(\beta,np) \cdot R_\beta(np,m\xi) \cdot \frac{so}{\sqrt{so^2+m^2\xi^2+n^2p^2}}\right] * h(np)\right\} d\beta,$$

$$s = -x\sin\beta + y\cos\beta. \tag{3}$$

This is the FDKHSFW scheme, where $\Delta$ is half the full fan angle of the central-scanning plane along the $t$ axis. The off-scanning plane projection data are still treated as they have the same redundancy. $\omega(\beta, np)$ is the discrete weighting coefficient, calculated based on the scanning plane geometry, and can be represented by Parker's weighting function or any other weighting function as long as it can make a smooth transition between the doubly and singly sampled regions to avoid discontinuities at the borders of these regions. Undoubtedly, FDKHSFW holds all the properties that the FDK full-scan scheme does.

For cone beam projection data off the scanning plane, however, it is impossible to obtain completely doubly sampled projections for a single circular orbit acquisition, even if projections are sampled over 360° [5]. In other words, the projection redundancy becomes less and less when projection rows get further away from the scanning plane. If the FDK algorithm had been directly applied to unweighted half-scan projection data, the reconstructed images would unavoidably have artifacts. One way to handle the weighting on the less redundancy projection row data away from scanning plane is proposed as follows:

$$\omega(\beta', np) = \begin{cases} \sin^2\left(\dfrac{\pi}{4}\dfrac{\beta'}{\Delta' - \tan^{-1}(np/so')}\right), & 0 \le \beta' \le 2\Delta' - 2\tan^{-1}\left(\dfrac{np}{so'}\right), \\ 1, & 2\Delta' - 2\tan^{-1}\left(\dfrac{np}{so'}\right) \le \beta' \le \pi - 2\tan^{-1}\left(\dfrac{np}{so'}\right), \\ \sin^2\left(\dfrac{\pi}{4}\dfrac{\pi + 2\Delta' - \beta'}{\Delta' + \tan^{-1}(np/so')}\right), & \pi - 2\tan^{-1}\left(\dfrac{np}{so'}\right) \le \beta' \le \pi + 2\Delta', \end{cases} \tag{4}$$

where

$$\beta' = \beta \cdot \frac{1}{\sqrt{1 + m^2\xi^2/so^2}}, \qquad so' = \sqrt{so^2 + m^2\xi^2}, \qquad \Delta' = \tan^{-1}\left(\frac{MO}{so'}\right). \tag{5}$$

$\beta'$ is the cone-weighting angle which will be described in the next section. $\beta'$ is dependent on the position of the row projection data in the $Z$ direction (rotation axis). $\Delta'$ is half of the titled fan angle that is adopted from Gullberg and Zeng [5]. Notice that when $m$ is zero, this weighting function is actually the Parker's weighting function for fan-beam.

By incorporating the cone-beam weighting function with FDK, FDKHSCW is obtained as follows:

$$f(x, y, z) = \int_0^{\pi+2\Delta} \frac{so^2}{(so - s)^2} \cdot \left\{\left[\omega(\beta', np) \cdot R_\beta(np, m\xi) \cdot \frac{so}{\sqrt{so^2 + m^2\xi^2 + n^2p^2}}\right] * h(np)\right\} d\beta, \qquad s = -x\sin\beta + y\cos\beta. \tag{6}$$

Please note that the projection data must be weighted prior to being filtered. Since FDKHSFW is the commonly acknowledged scheme for half-scan reconstruction, the requirement for FDKHSCW is that it should produce no more artifacts than FDKHSFW.

### 2.2. Further investigation of half-scan cone beam weighting

In a circular fan-beam half-scan, there are two redundant regions in the scanning plane in terms of the projection angle $\beta$. Figure 2 shows that the projecting ray data acquired in region I will have a conjugate ray data in region II. In these two regions, the projection ray data is wholly or partly redundant. If half of the full fan angle is $\Delta$ *degrees*, the half-scan range in terms of projection angle defined in the scanning plane is from 0° to 180° + 2Δ. The first and second redundant region is from 0° to 4Δ and from 180° − 2Δ to 180° + 2Δ, respectively. In the traditional FDK cone-beam half-scan scheme, all the row projection data are weighted by the same set of coefficients defined in the scanning plane because the row projection data away from the scanning plane are expected to have the same redundancy as those in the scanning plane.

The proposal of the circular cone beam half-scan weighting scheme is based on the idea that the weighting coefficients should be different for projection data in different rows, and for the row projection data furthest away from the scanning plane, it should be weighted less. As of this date, we have not seen any literature discussing this issue. We found that if we use $\beta' = \beta(1/\sqrt{1 + m^2\xi^2/so^2})$ as the weighting angle for different row projection data, then, the weighting coefficients in the first redundant region away from the scanning plane are not much different from those calculated in the scanning plane; the biggest difference is below 0.2 percent if $\Delta = 15°$ and the half cone angle is also 15°. On the other hand, when $\beta'$ is used as the weighting

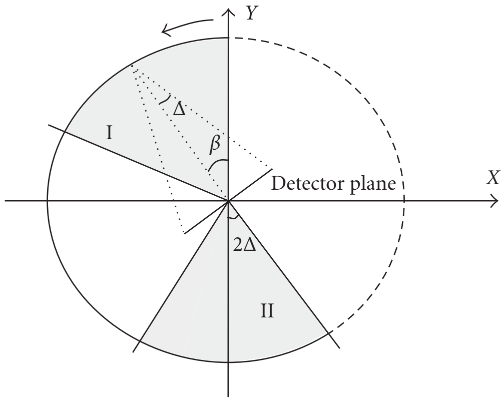

FIGURE 2: Illustration of redundant region in terms of projection angle in circular fan beam half scan.

angle in the second redundant region, the weighting coefficients away from the scanning plane behave obviously differently from those in the scanning plane and different from each other at the different rows, thus resulting in the compensation for the density drop in the place away from the scanning plane in the reconstruction image. The weighting angle $\beta'$ has two characteristics: first, it has row position dependence that is reflected by $m\xi$, indirectly connected to the cone angle information; second, it has less difference from $\beta$ when $\beta$ is in the first redundant region than when $\beta$ is in the second redundant region. Thus, it is beneficial to construct the cone angle dependent weighting coefficients in the second redundant region to achieve our scheme.

### 3. COMPUTER SIMULATION AND EVALUATION

In order to make computer simulation closer to the practical CBCT configuration, geometric parameters are set in terms of physical length (millimeter) rather than normalized units. The distances from the X-ray source to the iso-center of the reconstruction and to the detector are 780 mm and 1109 mm respectively. The full fan and cone angles are 30 degrees. The detector area is $595 \times 595\,\text{mm}^2$ and has a 512 by 512 matrix size. The voxel size is $0.816\,\text{mm}^3$. Cartesian coordinate $(X, Y, Z)$ is used to define the object, where $Z$ is the rotation axis. The sampling rate of projection angle is $0.8^\circ$ with the total number of projection images of 450 for full scan and 262 for half scan. The low contrast Shepp-Logan phantom was used (see [7] for geometrical parameters), all of its geometrical parameters are multiplied by 200 to simulate the physical length (millimeter) of the phantom.

### 3.1. The weighting coefficients distribution comparison of FDKHSCW and FDKHSFW

Based on the scanning geometrical parameters defined above, weighting coefficient distributions associated with FDKHSFW and FDKHSCW are compared by picking up $\beta = 46^\circ$ in the redundant region I and $\beta = 192^\circ$, as Figure 3 illustrates, in the redundant region II as described in Section 2.2.

### 3.2. Reconstruction comparison of FDKHSFW and FDKHSCW

Figure 4 shows the reconstructed sagittal image from different FDK schemes at $X = 0\,\text{mm}$ with the display window [1.005 1.05] and the profile comparison along the solid white lines in the phantom image (d). The ramp filter was used on the noise-free weighted projection data before backprojection.

### 3.3. Simulation on quantum noise contaminated projection data

In order to test the performance of this new scheme over the quantum noise that is commonly encountered in practical CBCT data acquisition, we generated quantum noise contaminated data. X-ray with 100 kVp was selected which corresponds to an effective photon fluence of $2.9972 * 10^7$ photons/cm$^2$ $\cdot$ mR [12]. The exposure level per projection was set to 4 mR, the total exposure levels for FDKFS and FDKHSCW are 1800 mR and 1048 mR, respectively. Figure 5 shows the reconstructed results under different noise levels and profile comparisons. Hamming window is used during filtering to suppress the noise.

### 4. DISCUSSIONS AND CONCLUSION

A new cone beam weighting scheme has been heuristically proposed for the FDK-based circular half-scan reconstruction (FDKHSCW) to correct the density drop artifact to a certain degree along the rotation axis inherited with original FDK algorithm for large cone angle. Computer simulation on the Shepp-Logan phantom with and without noise showed an improvement when using FDKHSCW over FDKFS and FDKHSFW in terms of the attenuation

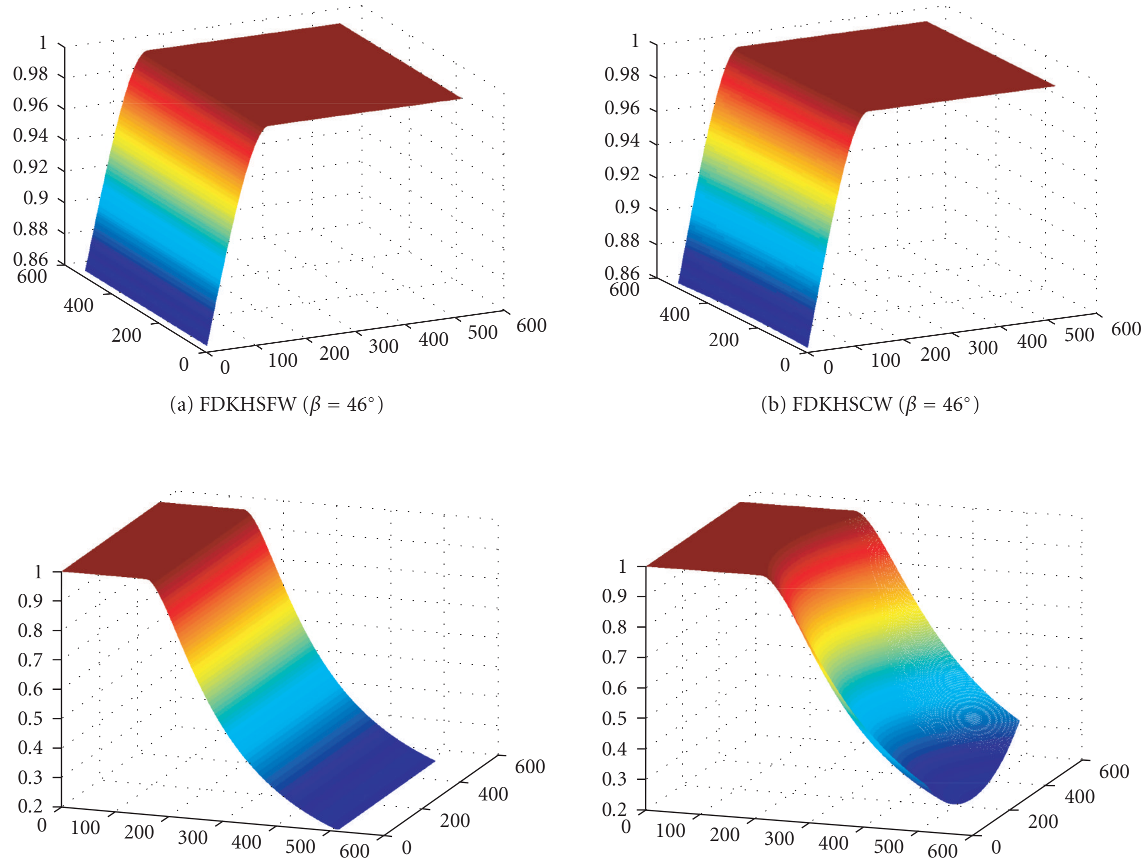

(a) FDKHSFW ($\beta = 46^\circ$)

(b) FDKHSCW ($\beta = 46^\circ$)

(c) FDKHSFW ($\beta = 192^\circ$)

(d) FDKHSCW ($\beta = 192^\circ$)

FIGURE 3: Weighting coefficients comparison between FDKHSFW and FDKHSCW when $\beta = 46^\circ$ and when $\beta = 192^\circ$ as shown in (a), (b) and (c), (d), respectively.

coefficient drop when the cone angle is large while maintaining the same visual image quality. FDKHSCW needs additional cone-beam weighting before filtering and only uses a scanning range of $[\beta, 180 - \beta - 2\Delta]$, where $\beta$ is the starting projection angle of X-ray, and $\Delta$ is half of the full fan angle; both of them are defined in the scanning plane. As soon as the starting angle is determined, each projection image can be processed (cone-beam weighting for half scan, pixel weighting inherited by FDK, and filtering). So, it will take less time to reconstruct an object in comparison to the full-scan scheme, a very desirable feature in practice. In addition, the half-scan scheme provides a flexibility to choose any starting point for reconstruction as long as the scanning range is guaranteed, another preferable feature for cone beam CT dynamic imaging. Based on the idea proposed by Silver [13], we can even conduct an extended half-scan scheme by making the scanning range larger than $180 - 2\Delta$ applying this new cone beam weighting function for better noise characteristic.

Our proposed circular cone-beam half-scan weighting scheme works better for low-contrast object. We can see from our simulation on Shepp-Logan phantom that the largest compensation is within 0.03 in terms of attenuation coefficient. We expect that FDKHSCW can show improvement in terms of intensity drop in the high-contrast phantoms, like a Defrise disk phantom. Yet, it is not as promising as in the low-contrast phantom.

Other proposed modified FDK methods called T-FDK and FDK-SLANT [14, 15] also corrected the attenuation coefficient drop to some extent along the rotation axis inherited in FDK with a larger cone angle. There is a difference between these methods and FDKHSCW. Although the results of these methods showed similar correction to FDKHSCW, FDK-SLANT and T-FDK need to be parallel rebinned from cone beam data. That means the filtering portion would not start until the whole set of data acquisition and parallel resorting procedures are completed and then followed by backprojection for image reconstruction. FDKHSCW possess the advantage that once a 2D projection data is acquired, the filtering portion can start and be immediately followed by backprojection. As long as the gantry speed and readout rate is high enough, this scheme can provide almost real time monitoring when continuous dynamic imaging is conducted. Wang [16] developed a weighting scheme for cone beam full circular-scan

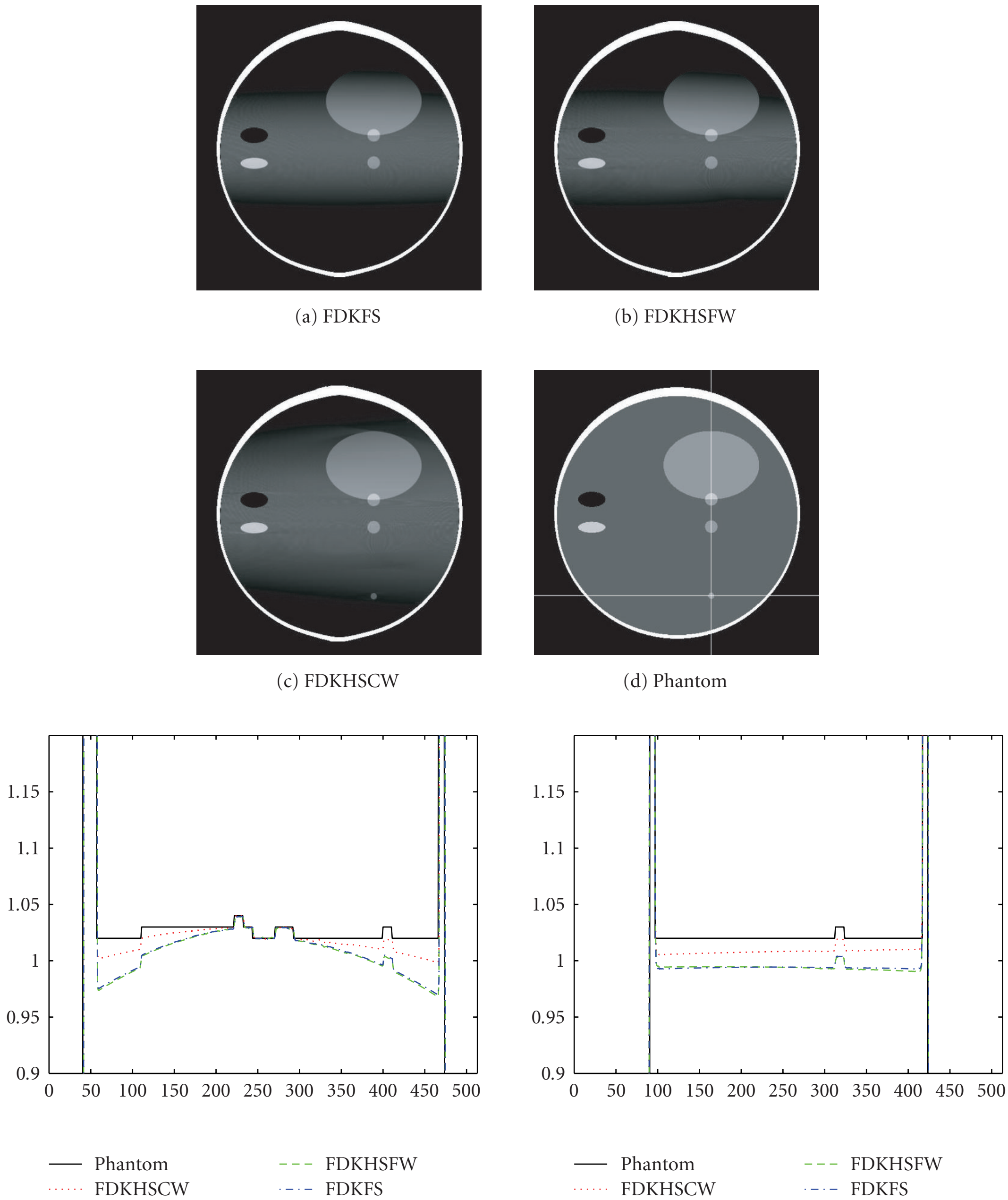

(a) FDKFS (b) FDKHSFW

(c) FDKHSCW (d) Phantom

(e) (f)

Figure 4: Reconstructed sagittal image from different FDK schemes at $X = 0$ mm and respective line profile comparison in (e) and (f) along the solid vertical and horizontal white line shown in (d).

reconstruction on a displaced detector array without rebinning the projection data for reconstruction. As for the redundant area, our scheme can be applied to this algorithm by adjusting the weighting conditions in the scanning range.

Recently a new circular 3D weighting reconstruction algorithm [17] was proposed to reduce cone beam artifact based on the investigation on the data inconsistency between a direct ray and its conjugate rays. The basic idea is to have filtered projection data multiplied by correction coefficients that are cone beam geometrical dependent during the backprojection. But the artifact it corrects is not what FDKHSCW tries to correct here, namely attenuation coefficient drop. However, it is worth trying to combine these two schemes for future evaluation.

In conclusion, by incorporating a new cone beam weighting scheme, a new FDK-based heuristic half-scan approximate algorithm for circular trajectory has been proposed based on flat panel detector, and the numerical simulation demonstrated its feasibility.

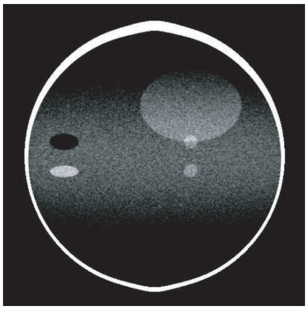

(a) FDKFS (1800 mR)

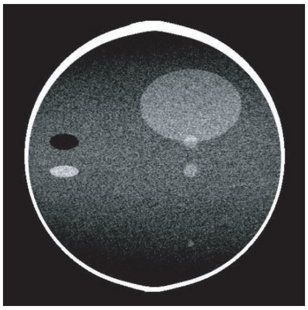

(b) FDKHSCW (1048 mR)

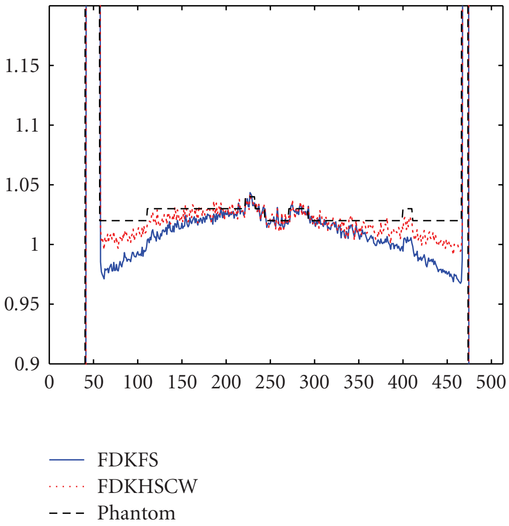

(c) Zoomed profile comparison along the vertical line in Figure 3(d)

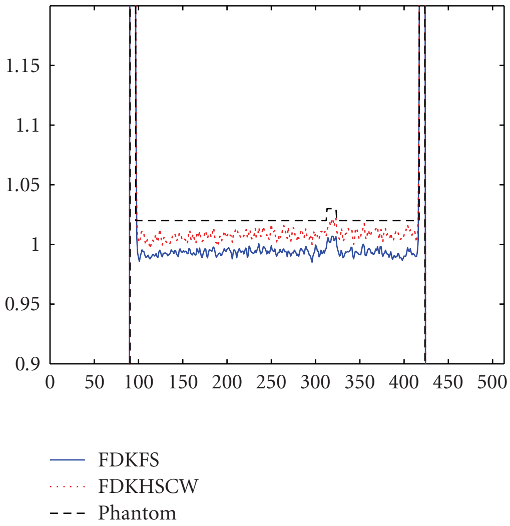

(d) Zoomed profile comparison along the horizontal line in Figure 3(d)

Figure 5: (a) FDKFS with total exposure level of 1800 mR. (b) FDKHSCW with total exposure level of 1048 mR. (c), (d) Profile comparison between FDKFS, FDKHSCW, and phantom along the solid vertical and horizontal lines in Figure 4(d).

### ACKNOWLEDGMENTS

The authors thank the anonymous reviewers for their helpful comments. This project was supported in part by NIH Grants 8 R01 EB002775, R01 9 HL078181, and 4 R33 CA94300.

### REFERENCES

[1] Y. Liu, H. Liu, Y. Wang, and G. Wang, “Half-scan cone-beam CT fluoroscopy with multiple x-ray sources,” *Medical Physics*, vol. 28, no. 7, pp. 1466–1471, 2001.

[2] K. Taguchi, “Temporal resolution and the evaluation of candidate algorithms for four-dimensional CT,” *Medical Physics*, vol. 30, no. 4, pp. 640–650, 2003.

[3] G. Wang, Y. Liu, T. H. Lin, and P. C. Cheng, “Half-scan cone-beam x-ray microtomography formula,” *Scanning*, vol. 16, no. 4, pp. 216–220, 1994.

[4] S. Zhao and G. Wang, “Feldkamp-type cone-beam tomography in the wavelet framework,” *IEEE Transactions on Medical Imaging*, vol. 19, no. 9, pp. 922–929, 2000.

[5] G. T. Gullberg and G. L. Zeng, “A cone-beam filtered backprojection reconstruction algorithm for cardiac single photon emission computed tomography,” *IEEE Transactions on Medical Imaging*, vol. 11, no. 1, pp. 91–101, 1992.

[6] F. Noo and D. J. Heuscher, “Image reconstruction from cone-beam data on a circular short-scan,” in *Medical Imaging 2002: Image Processing*, vol. 4684 of *Proceedings of SPIE*, pp. 50–59, San Diego, Calif, USA, February 2002.

[7] S. W. Lee and G. Wang, “A Grangeat-type half-scan algorithm for cone-beam CT,” *Medical Physics*, vol. 30, no. 4, pp. 689–700, 2003.

[8] P. Grangeat, “Mathematical framework of cone beam 3D reconstruction via the first derivative of the Radon transform,” in *Mathematical Methods in Tomography*, G. T. Herman, A. K. Luis, and F. Natterer, Eds., Lecture Notes in Mathematics, pp. 66–97, Springer, Berlin, Germany, 1991.

[9] D. L. Parker, “Optimal short scan convolution reconstruction for fan beam CT,” *Medical Physics*, vol. 9, no. 2, pp. 254–257, 1982.

[10] G. Wang, C. R. Crawford, and W. A. Kalender, “Multirow detector and cone-beam spiral/helical CT,” *IEEE Transactions on Medical Imaging*, vol. 19, no. 9, pp. 817–821, 2000.

[11] I. A. Feldkamp, L. C. Davis, and J. W. Kress, "Practical cone-beam algorithm," *Journal of the Optical Society of America A*, vol. 1, no. 6, pp. 612–619, 1984.
[12] Y. Zhang, R. Ning, D. Conover, and Y. Yu, "Image noise due to quantum fluctuations in flat-panel detector based cone-beam CT imaging," in *Medical Imaging 2005: Physics of Medical Imaging*, vol. 5745 of *Proceedings of SPIE*, pp. 656–663, San Diego, Calif, USA, February 2005.
[13] M. D. Silver, "A method for including redundant data in computed tomography," *Medical Physics*, vol. 27, no. 4, pp. 773–774, 2000.
[14] M. Grass, Th. Kōhler, and R. Proksa, "3D cone-beam CT reconstruction for circular trajectories," *Physics in Medicine and Biology*, vol. 45, no. 2, pp. 329–347, 2000.
[15] H. Turbell, "Cone-beam reconstruction using filtered back-projection," Linkoeping Studies in Science and Technology Dissertation 672, Linkoeping University, Linkoeping, Sweden, 2001.
[16] G. Wang, "X-ray micro-CT with a displaced detector array," *Medical Physics*, vol. 29, no. 7, pp. 1634–1636, 2002.
[17] X. Tang, J. Hsieh, A. Hagiwara, R. A. Nilsen, J.-B. Thibault, and E. Drapkin, "A three-dimensional weighted cone beam filtered backprojection (CB-FBP) algorithm for image reconstruction in volumetric CT under a circular source trajectory," *Physics in Medicine and Biology*, vol. 50, no. 16, pp. 3889–3905, 2005.

---

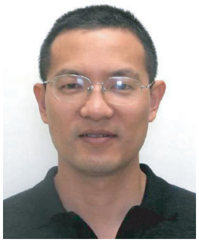

**Dong Yang** received his M.S. degree in biomedical engineering in 1998 from Chongqing University, China. He got the M.S. degree in electronic and computer engineering in 2004 from University of Rochester, USA. He is now a Ph.D. candidate in the Department of Electrical and Computer Engineering of the University of Rochester. His research work is in the flat panel-based cone beam CT reconstruction implementation and clinical application, and medical imaging processing.

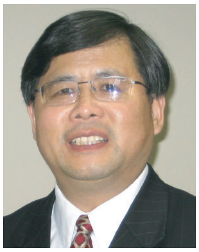

**Ruola Ning** received a B.S. degree in electronic physics from Zhongshan University in China in 1982, an M.S. degree in electrical engineering in 1986, and a Ph.D. degree in electrical engineering in 1989 from the University of Utah. He is a Member of both SPIE and AAPM. Since 1989, he has been on the Faculty of the University of Rochester. He is an ABR certified medical physicist and is the author of more than 80 referred journal articles and proceedings. He is currently a Professor of imaging sciences (the formal radiology), biomedical engineering, electrical & computer engineering, radiation oncology, and oncology at the University of Rochester, where his research interests include the development of cone beam CT imaging, cone beam breast CT, cone beam CT angiography, functional cone beam CT, cone beam reconstruction algorithms, three-dimensional medical imaging, pattern recognition, and image-based explosive detection.

